# Blue shift of CdSe/ZnS nanocrystal-labels upon DNA-hybridization

**DOI:** 10.1186/1477-3155-6-7

**Published:** 2008-05-19

**Authors:** Jürgen Riegler, Franck Ditengou, Klaus Palme, Thomas Nann

**Affiliations:** 1 Fraunhofer Institute for Interfacial Engineering and Biotechnology, Nobelstrasse 12, 70569 Stuttgart, Germany; 2 Institute of Biology II/Botany, Faculty of Biology, Albert-Ludwig University Freiburg, Schänzlestr. 1, 79104 Freiburg, Germany; 3 School of Chemical Sciences and Pharmacy, University of East Anglia, Norwich Research Park, Norwich NR4 7TJ, UK

## Abstract

Luminescence color multiplexing is one of the most intriguing benefits, which might occur by using semiconductor Quantum Dots (QDs) as labels for biomolecules. It was found, that the luminescence of QDs can be quenched, and replaced by a luminescence peak at approximately 460 nm on hybridization with certain regions of *Arabidopsis thaliana *tissue. This effect is site selective, and it is unclear whether it occurs due to an energy transfer process, or due to quenching and scattering of the excitation light. The article describes methods for phase-transfer of differently coloured, hydrophobically ligated QDs, coupling of DNA strands to the QD's surface, and hybridization of the labelled DNA to different cell types of *Arabidopsis thaliana*. The reason for the luminescence blue-shift was studied systematically, and narrowed down to the above mentioned causes.

## Background

Fluorescence is a widely used tool in biology to study the complexity and dynamics of biological processes. Compared to conventional organic dye molecules, fluorescent semiconductor nanocrystals (QDs) have several promising advantages. They can be excited by a broad range of wavelengths from UV up to their individual absorption edge, and they have narrow, tuneable emission spectra, which can be well resolved over the same spectral range. Moreover, in contrast to most organic fluorophores they are highly resistant to chemical and metabolic degradation and have a higher photobleaching threshold [1-5]. The challenges for using QDs in biological studies include designing hydrophilic QDs with surface chemistry well adapted to different biological applications. Surface modified QDs should be luminescent with optical properties not differing from the unmodified QDs [6-10].

Here we report the preparation of water-soluble CdSe/ZnS QDs, which have been surface modified for versatile and selective coupling of biological probes and subsequent specific labeling of cells. Because of the wide emission range, narrow spectral linewidth, brightness, and the adjustable, size dependent emission wavelengths of these QDs, they are expected to be a good choice for multiplex-imaging. Theoretically our CdSe/ZnS QDs should allow labeling of several different probes and imaging of up to eight different biological molecules in the visible range of the spectrum [11,12]. We demonstrate that synthetic oligonucleotides can be efficiently covalently linked to these QDs. We further show that they can be used for subsequent analysis of expressed genes by *in situ *hybridisation experiments. The technique was successfully applied to detect transcripts in the plant *Arabidopsis thaliana*, a fully sequenced model organism [13].

## Materials and methods

### Preparation of QDs

CdSe/ZnS core/shell QDs were prepared according to a method published previously [14]. Briefly, cadmium stearate and trioctylphosohine-selenid (TOP-Se) were reacted at temperatures above 200°C by fast injection of TOP-Se into a mixture of trioctylphosphine-oxide (TOPO) and cadmium stearate. The CdSe-cores were passivated and annealed by growing a shell of two additional monolayers of ZnS on their surface. Diethylzinc and hexamethyldisilathian were reacted for 12 hours with the CdSe-cores at 160°C in the presence of TOPO and TOP, again. The core/shell particles obtained were repeatedly washed with methanol, and re-dispersed in chloroform. Finally the particles were stored in 50 ml chloroform as stock solution.

### Phase transfer and conjugation of DNA

Phase transfer of the CdSe-QDs to water phase and conjugation to oligonucleotides was carried out by double ligand exchange by modifying the procedure of Mirkin et al. [15]. In a first step the TOPO on the surface of the particles was exchanged with mercaptopropionic acid (MPA). 10 ml (0.1 molar) of a solution of MPA in dimethylformamide (DMF) were added to solid QDs, precipitated out of 5 ml of each stock solution by addition of 10 ml methanol and subsequent centrifugation. To complete the ligand exchange, the QD solutions were incubated for 12 hours at 80°C. To precipitate MPA-QDs, 100 μg of dimethylaminopyridine (DMAP) were added to each sample, followed by centrifugation at 10,000 g. The supernatants with unincorporated MPA and DMPA were discarded and the pellets containing QDs were dispersed in 1 ml of specific thionylated oligonucleotides in water. The slightly colored solutions were incubated for additional 24 hours at room temperature to partially exchange MPA against the thionylated oligonucleotides. Finally the QD-oligonucleotide conjugates were yielded by adding 3 M NaCl and dialysis the solution against water for 72 hours while the receiver was three times renewed. Five different antisense specific QD-oligonucleotide conjugates were prepared with emission-wavelengths at 543 nm (At5g05600-5'gcatgcatgaaggcaaatcatcctttgaaaattcaaaatataaatgattgtacacatatacaagtcagacgtaatatc3'), 563 nm (At1g73590-5'agaaagattagaggctctaggggttaagcacaaggagggggacataa3'), 598 nm (At5g47910-5'cagagatctatacaaataaacacccgtaaggttactgtattagttgatagagaaaaaataaccgctctc3'), 610 nm (At5g50960 5'cgtcgacttgagacttctcgaagggaatttttcgtttatatgtgaaactctctgcttatggcggcg'), and 653 nm (At2g24200-5' ctgcacgactaaaacaaagtaccactttattcaacttttgacgattttacttttcataac) respectively. Same oligonucleotides labeled with fluorescein (Genedetect-New Zealand) were used as controls.

### In situ hybridization

*Arabidopsis thaliana *floral meristems of 24 days old plants were fixed with 4% paraformaldehyde in PBS (pH 7.3) and embedded in paraffin. 7 μm tissue sections mounted on SuperFrost^® ^slides (Carl Roth, Germany) were used for *in situ *hybridization. *In situ *hybridization was performed as follows. SuperFrost^® ^slides holding sectioned paraffin embedded *Arabidopsis thaliana *inflorescence were de-waxed by placing them in 3 changes of histoclear (PLANO GmbH) for 3 minutes each, followed by 2 changes of histoclear/ethanol (2:1 and then 1:2) followed by 3 changes of 100% ethanol for 3 minutes each. Tissues were re-hydrated in 95%, 70%, 50% and 30% ethanol, for 2 minutes each. Slides were incubated in 0.2 M HCl for 20 min at room temperature. Slides were washed 2 × 5 min in PBS and tissue were then permeabilized for 20 min at 37°C with TE buffer (20 mM Tris-HCl pH 7.5, 2 mM CaCl_2_) containing 20 μg/ml of RNAse free Proteinase K (Roche Diagnostics, Basel, Switzerland). The enzymatic reaction was stopped by incubating slides in 0.2 mg/ml glycine. After two rinses in PBS, tissues were carefully overlaid with prehybridization buffer [50% formamide deionised, 2× SSC (from 20× SSC, sodium chloride and 300 mM trisodium citrate, pH 7.0), 10 mg/ml yeast tRNA, 2% dextransulfate, 10 mg/ml Poly A, 10 mg/ml ssDNA, 100 mM DTT, 50× Denhardts] and incubated in a humid sealed chamber at 37°C for 2 hours. After 5 minute rinse in 2× SSC, slides were overlaid with prehybridization buffer supplemented with labelled oligonucleotides (25 ng/ml; 1/400 dilution) and incubated overnight at 37°C. Post hybridization washes were done as follows. A quick wash in 1× SSC (10 mM DTT) at RT, 2 × 15 min in 1× SSC (10 mM DTT) at 55°C, 2 × 15 min in 0.5× SSC (10 mM DTT) at 55°C and 1× in 0.5× SSC (10 mM DTT) at RT. For sample processing the In SituPro robot (Intavis AG) was used.

### Microscopy

Plant sections were imaged using a laser scanning microscope (LSM) (Zeiss LSM 510 META). QD-labeled oligonucleotides were excited with a laser beam in the UV range (405 nm excitation wavelength) and the full visible emission spectrum was recorded. This allowed to precisely differentiate expected emission wavelength from background emission. This method also allowed detection of any shift in QD emission wave length.

### Spectroscopy

Photoluminescence (PL) and absorption spectra were recorded on a J&M TIDAS diode array spectrometer using standard quartz cuvettes. QD spectra were recorded in aqueous buffer solution.

## Results and Discussion

### Generalized approach for QD labeling of oligonucleotides

**Figure 1:**
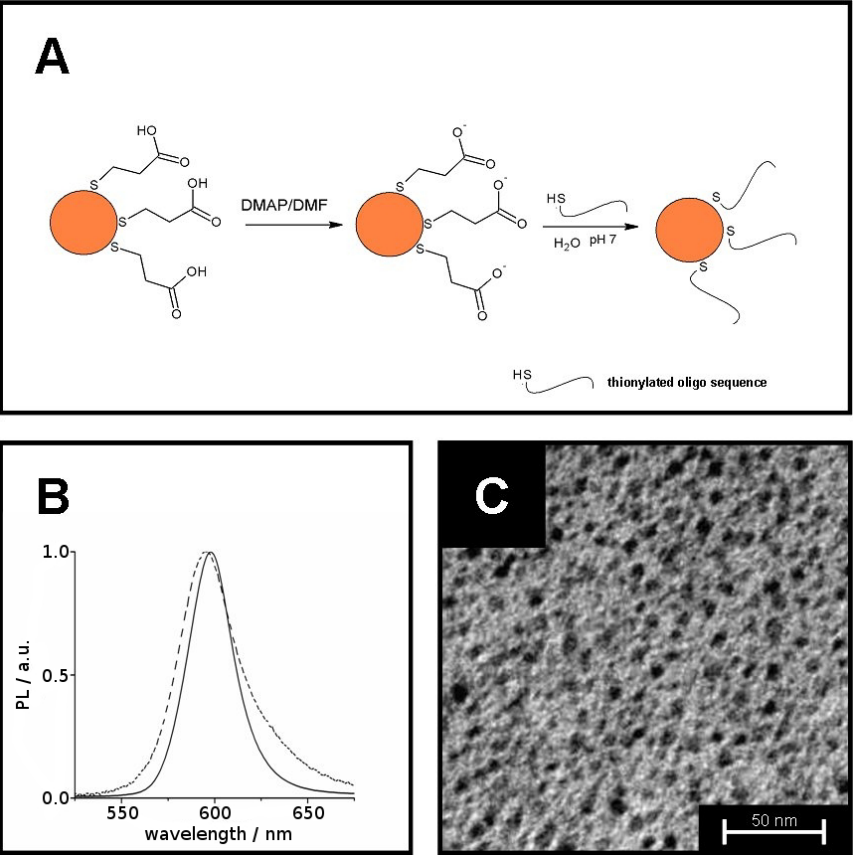
**A) Scheme of the derivatization of the QDs by double ligand exchange.** Surface TOPO is replaced with MPA followed by the partial replacement of the MPA with thionylated oligonucleotides. B) Luminescence spectra of TOPO-QDs (solid line) and their corresponding oligonucleotide derivatives respectively (dashed line). Original emission-wavelength is slightly shifted to the red by TOPO replacement. C) TEM-picture of QD-oligonucleotide derivatives. There are no agglomerates observed after surface modification.

We and others have already reported the chemical synthesis of CdSe/ZnS core/shell QDs and their optical properties [14,16]. Here we have surface modified these QDs, and developed protocols for covalent coupling of these particles to oligonucleotides. Figure 1(A) shows schematically the steps occurring during the exchange process on the QD-surface. The QDs were first transferred to the water-phase, then surface-ligands (TOPO) were completely exchanged against MPA. In a second step the MPA was partially exchanged against the thionylated oligonucleotide, similar to a previously published procedure [15]. Figure 1(B) shows the emission spectra of TOPO capped QDs and of the corresponding oligonucelotide-derivatized QDs respectively. The maximum of emission was slightly shifted to the red by about 5 nm from 593 nm to 598 nm during the ligand exchange process. This emission wavelength shift was observed in all exchange reactions. In accordance with the literature, the red shift was taken as a hint for the successful ligand exchange [15]. Our QD-oligonucleotide conjugates were colloidally stable in water for three weeks at room temperature, before they started to agglomerate and precipitate. This was probably due to desorption of the ligands, changes of surface properties and alteration of charge density and quality. Figure 1(C) shows a transmission electron microscope (TEM) micrograph of oligonucleotide modified QDs directly after ligand exchange. This picture clearly shows the absence of larger agglomerates and thus proves the excellent dispersibility of the conjugates.

### In situ hybridization using QD-oligonucleotide conjugates

**Figure 2:**
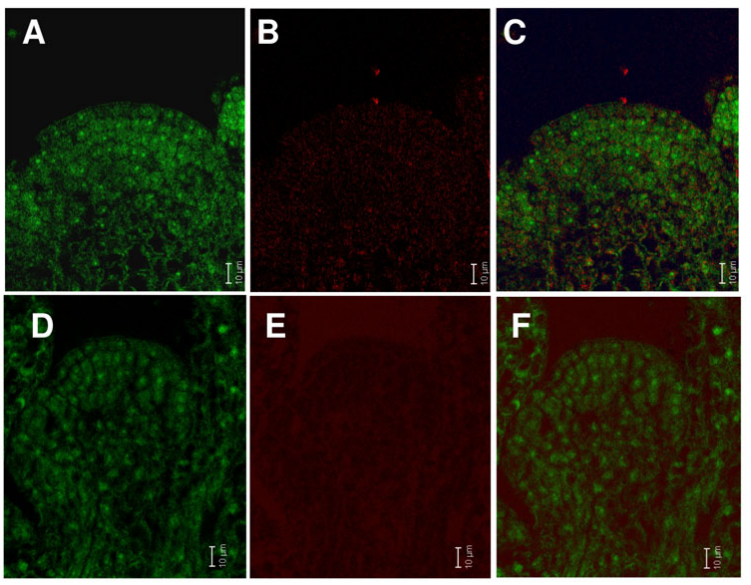
**A-C) AtPIN1 anti sense oligonucleotide linked to QD.** Specific signal observed in epidermal and meristematic cells. A) and B) display separated channels. D-F) AtPIN1 anti sense oligonucleotide linked to fluorescein. Fluorescence is observed in epidermal and meristematic cells. D) and E) depict separated channels. AtPIN1 expression for both probes is in agreement with previously reported AtPIN1 mRNA *in situ *localization signals [16].

In a first experiment, we used QD-oligonucleotide conjugates with an emission-wavelength of 563 nm for *in situ *detection of AtPIN1 (At1g73590) mRNA. We used laser scanning confocal microscopy to monitor the signals in fixed floral tissue sections. Figure 2(C) shows a tangential section through an Arabidopsis flower meristem. The bright green fluorescence corresponds to QD-oligonucleotide specific signals, while the red fluorescence visible at the lower border region corresponds to auto-fluorescence typically seen in these cells. Figure 2A) and 2B) show the separated channels. Specific signals are very strong indicating a good signal to noise ratio and specific binding of the QD-oligonucleotide to the target mRNA and no apparent binding of the probe to other cellular components. Compared to the signals visible in epidermal and meristematic cells using the same oligonucleotide labeled with fluorescein, the apparent large size of the QD-oligonucleotide does not modify the hybridization kinetics of the oligonucleotide with its complementary mRNA. (Figure 2D–F). mRNA patterns for both probes are well in agreement with previously reported AtPIN1 mRNA *in situ *localization signals [17]. In order to confirm the specificity of the QD-oligonucleotide signal we further performed control experiments using oligonucleotides in sense orientation which should give no signal. As expected we did not observe any specific signal for both fluorescein and QD labeled oligonucleotides. This clearly shows that our QD-oligonucleotides are usful for biological applications and able to specifically hybridize to their target sequences. QD-oligonucelotides were stable and could be used repeatedly many times demonstrating the specificity of the chemical and physical properties of the QD-oligonucleotides and the robustness our *in situ *assay conditions (data not shown).

### Generalized approach for multiparametric labeling

Simultaneous detection of several different biomolecules in the cellular context is essential for addressing many biological questions. In order to establish multiparametric analysis of mRNAs, five different oligonucleotides were covalently linked to QDs with distinguishable emission wavelengths. The spectral properties of these QD-oligonucleotide conjugates were as expected.

**Figure 3:**
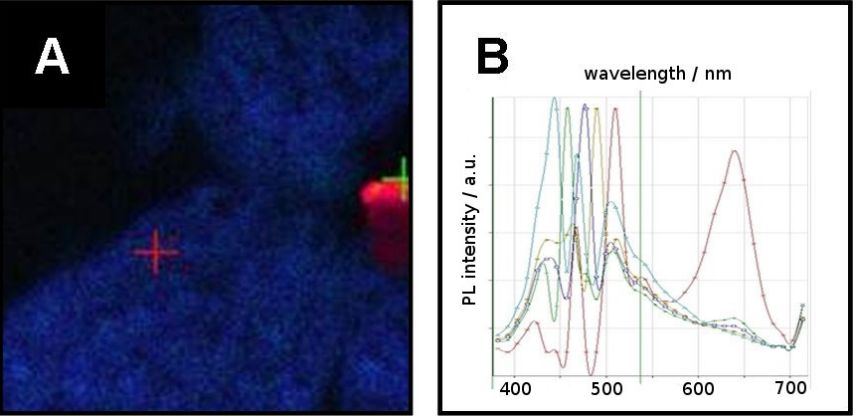
**A) LSM-picture of selectively labeled Arabidopsis tissue.** The tissue was hybridized with five different QD-oligonucleotides of distinguishable emission wavelength. The emission of QD-labels was shifted to the strong blue. This shift appears only after hybridization. Unreacted, agglomerated QD-oligonucleotides show their initial emission behavior (red area on the right border of the picture). B) Spectral analysis of picture A. The wavelength of all hybridized QD-oligonucleotides was shifted to 460 nm. The emission of some non hybridized QD-oligonucleotides could be found at 598 nm.

These QD-oligonucleotides were used for in situ hybridization to detect individual mRNAs in separate *in situ *hybridizations as well as by combining all five QD-oligonucleotides in a single *in situ *hybridization experiment. Whereas *in situ *hybridization of each single QD-oligonucleotide resulted in specific signals there was only a blue emission observed after hybridization of a 1:1:1:1:1 mixture of all five QD-oligonucleotides (Figure 3A).

This blue emission does not correspond to any known fluorophore present in the experimental setup. Figure 3b shows the corresponding spectral analysis. After blue-shifting, the luminescence maximum was found at about 460 nm independent of the initial luminescence of the QD-label (not shown in Figure 3). It was observed, that the initial luminescence of the QDs decreased, while the luminescence peak at around 460 nm increased. In other words: the luminescence did not shift gradually to the blue, but "leaped" to the blue. The shapes of the luminescence spectra around 460 nm are similar to those of the original QDs.

**Figure 4:**
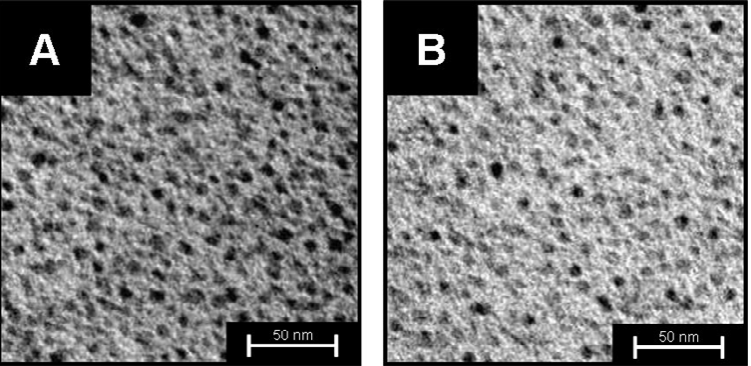
**TEM-micrograph of QD-oligonucleotides before (A) and after (B) hybridization process respectively.** Both pictures are showing non-agglomerated QDs with a diameter of around 3 nm. Samples were prepared by immersion and subsequent drying of carbon-film covered copper grinds in aqueous dispersions of sample A and B.

Figure 4 displays TEM micrographs of QD-oligonucleotide conjugates before (A) and after (B) hybridization. The sizes of QDs in both pictures were to be found at about 3 nm, indicating, that the observed blue shift was not caused by the reduction in size of the QDs.

**Figure 5:**
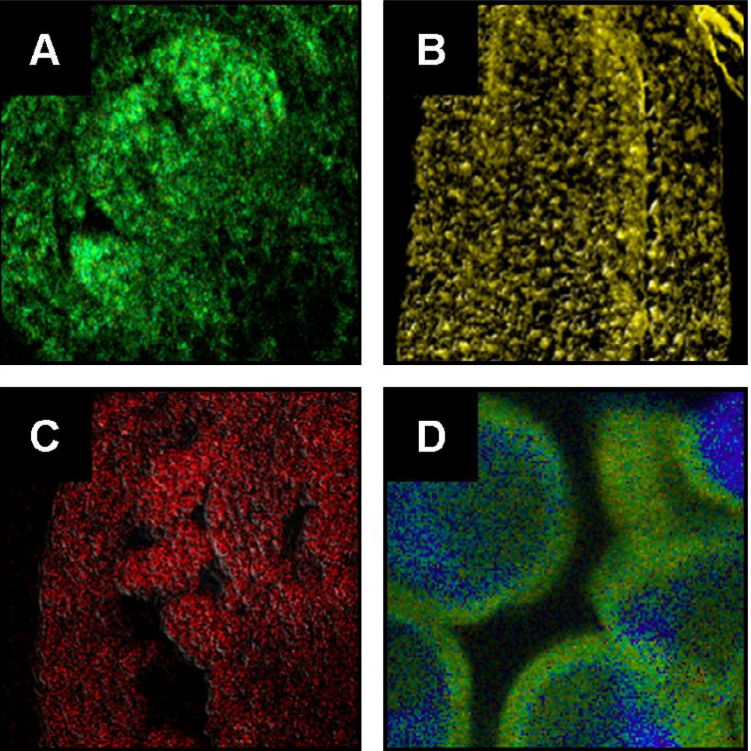
**Several LSM-pictures of Arabidopsis tissues, which were hybridized with distinguishable QD-oligonucleotides.** In blossom (A, C), and leaf (B) specific hybridization and labeling took place while in the case of pollen (D) the QD-emission was strongly shifted after hybridization. The luminescence of unspecific bounded QD-oligonucleotides on the surface of the pollen was not shifted.

The "blue shift" was directly related to the hybridization process. Figure 3(A) also shows some aggregated (non-hybiridized) QDs (red), which kept their original luminescence properties (spectrum depicted in 3 (B)). Furthermore the blue-shift was related to the biological sample and the region within the sample. Figure 5A to 5C depict non shifted luminescence of different QD-labels after specific hybridization to flower (A,C) and leaf tissue (B). Figure 5(D) shows the blue-shifted emission of the QD-label found after hybridization in pollen. Remarkably, the luminescence of non specifically bound QD-oligonucleotide conjugates on the surface of the pollen (figure 5D) was not affected.

The observations reported above rule out the obvious explanation for blue-shift of QD-emission, namely photo corrosion. Therefore the blue shift seems to be caused by an energy-transfer process that is related to hybridization or by selective quenching of the QD luminescence and light scattering [18]. So far, it was not possible to explain this phenomenon fully. Nevertheless, it is an interesting observation which may pave the way for potential QD-based *in-vivo *sensors.

In this paper we report the specific labeling and imaging of Arabidopsis tissue sections by the use of distinguishable QD-oligonucleotide conjugates. After hybridization, regions within the Arabidopsis flower like pistil, leaves or pollen could be depicted respectively on a cellular level by LSM. Surprisingly, a strong blue-shift together with a reduction of luminescence intensity of the initial QD-fluorescence was observed. This remarkable blue-shift does not originate from the oxidative corrosion of QDs as it appears only after hybridization of coupled QDs. Finally, the origin of the blue shift could not be clarified within the presented work. Most likely, the blue-shift is caused by selective luminescence quenching and light scattering at hybridized QDs. The discussed blue-shift was not observed with organic fluorophores. Even though this effect is not fully understood yet, it might be potentially interesting for *in-vivo *molecular imaging, because of its sensitivity against the biological microenvironment.

## Authors' contributions

JR and FD contributed equally to this work. JR prepared and derivatized the Quantum Dots, FD carried out the biological experiments. KP and TN conceived of the study, and participated in its design and coordination. All authors read and approved the final manuscript.
